# DNA methyltransferases and stress-related genes expression in zebrafish larvae after exposure to heat and copper during reprogramming of DNA methylation

**DOI:** 10.1038/srep34254

**Published:** 2016-10-12

**Authors:** Jennifer Dorts, Elodie Falisse, Emilie Schoofs, Enora Flamion, Patrick Kestemont, Frédéric Silvestre

**Affiliations:** 1 Research Unit in Environmental and Evolutionary Biology, University of Namur, Rue de Bruxelles 61, B-5000 Namur, Belgium

## Abstract

DNA methylation, a well-studied epigenetic mark, is important for gene regulation in adulthood and for development. Using genetic and epigenetic approaches, the present study aimed at evaluating the effects of heat stress and copper exposure during zebrafish early embryogenesis when patterns of DNA methylation are being established, a process called reprogramming. Embryos were exposed to 325 μg Cu/L from fertilization (<1 h post fertilization - hpf) to 4 hpf at either 26.5 °C or 34 °C, followed by incubation in clean water at 26.5 °C till 96 hpf. Significant increased mortality rates and delayed hatching were observed following exposure to combined high temperature and Cu. Secondly, both stressors, alone or in combination, significantly upregulated the expression of *de novo* DNA methyltransferase genes (*dnmt3*) along with no differences in global cytosine methylation level. Finally, Cu exposure significantly increased the expression of metallothionein (*mt2*) and heat shock protein (*hsp70*), the latter being also increased following exposure to high temperature. These results highlighted the sensitivity of early embryogenesis and more precisely of the reprogramming period to environmental challenges, in a realistic situation of combined stressors.

In eukaryotes, DNA methylation is an important epigenetic mechanism involved in the regulation of gene expression and appears to be crucial for proper development of organisms^1^. DNA methylation occurs primarily through the transfer of a methyl group to the 5-position of cytosine residues in a CpG dinucleotide context and is catalyzed by a group of enzymes, the DNA methyltransferases (DNMTs). In mammals, there are three enzymatically active DNMTs, namely DNMT1, DNMT3a and DNMT3b. DNMT1 is maintaining methylation after each cell division, whereas DNMT3a and DNMT3b are two functionally related proteins that are responsible for *de novo* methylation during development and differentiation^2^. Due to genome duplication, zebrafish (*Danio rerio*), a well-established model in toxicology and developmental biology, has 8 different *dnmt* genes, of which 6 are most likely *de novo* DNMTs as they are most similar to the mammalian DNMT3 members^3^.

Recent studies in mammals, fish, and some invertebrate species have demonstrated that a variety of environmental stimuli, including exposure to contaminants, physiological stress and nutritional deficits, alters DNA methylation at either a global level or on selected genes^4^,^5^,^6^. In zebrafish, previous studies have primarily focused on DNA methylation changes triggered by environmental pollutants, such as arsenic, benzo[*a*]pyrene, 17α ethinylestradiol and methylmercury, in embryos, larvae or adults^7^,^8^. It has been further suggested that the DNA methylome is most susceptible to environmental challenges during early embryogenesis and particularly when patterns of DNA methylation are being established, a process called reprogramming^6^. Zebrafish is one of the few non-mammalian vertebrates in which DNA methylation reprogramming has been investigated. Significant differences in genome DNA methylation status have been found in zebrafish early embryos and germ cells. The oocyte DNA is globally hypomethylated compared to the one of sperm. After fertilization, zebrafish exhibit moderate bulk DNA demethylation, with *de novo* methylation starting at the 32-cell stage at 1^3/4^ h post-fertilization (hpf). The methylation level is then restored by sphere stage at 4 hpf to levels observed in sperm^9^,^10^,^11^,^12^. It is also noteworthy that in zebrafish the paternal DNA methylation pattern is maintained throughout early embryogenesis while the relatively hypomethylated maternal DNA is reprogrammed to a pattern similar to that of the sperm (i.e., zebrafish inherit the DNA methylome from sperm)^11^,^12^. Currently, alterations in DNA methylation due to environmental exposures during reprogramming have received little attention. Martin *et al*. reported severe phenotypical changes and lower global DNA methylation levels in zebrafish exposed to 5-azacytidine, a demethylating agent, from 1 to 24 hpf^13^. Shorter windows of exposure from 1 to 4 hpf appeared to be critical and resulted in similar results, whereas exposure from 6 to 24 hpf showed almost no effects on morphology. Interestingly, this sensitive early stage window corresponds with the dynamic demethylation and remethylation events that occur during development and indicates that this time window must be included in toxicity testing^7^. More recently, McGee *et al*. exposed zebrafish embryos to different flame retardant chemicals during early development. They reported a critical window of exposure from 0.75 hpf to 2 hpf, which induced mortality and malformations at 96 hpf and delayed remethylation of the zygotic genome^14^.

Accordingly, the present study aimed at evaluating the effects of two widespread environmental stress factors that frequently co-occur in aquatic systems, i.e., temperature and copper exposure, during the DNA methylation reprogramming, a presumed sensitive period of zebrafish development. Copper (Cu) is an essential micronutrient for all organisms, but, in excess, it can cause detrimental effects on fish from the cellular to population level^15^. In zebrafish, Johnson *et al*. reported that exposure of embryos to a range of copper concentrations from 50 to 1000 μg/L resulted in mortality, hatching inhibition, impairment of larval development and lateral line dysfunction^16^. It is also admitted that metal toxicity generally increases with rising temperature in aquatic species^17^,^18^. For instance, rising temperature was found to increase nickel toxicity in zebrafish embryos^19^, copper susceptibility in fathead minnow *Pimephales promelas*^20^ and cadmium toxicity in the European bullhead *Cottus gobio*^21^. Besides, although epigenetic changes following exposure to multiple stressors remain to date unknown, several studies suggested that thermal stress^22^,^23^,^24^ and metals exposure^4^,^25^,^26^ may result in an altered DNA methylation pattern. For instance, Zhou *et al*. demonstrated that exposure to copper, zinc, lead, cadmium or a metal mixture for 48 h increased global DNA methylation in the liver of the goldfish *Carassius auratus*^27^.

In order to test whether the reprogramming event is sensitive to the effects of a combination of environmental stressors, zebrafish embryos were exposed to Cu from fertilization (before complete hardening of the protective chorion) to 4 hpf (sphere stage) at either 26.5 °C or 34 °C, followed by incubation in clean water at 26.5 °C to 96 hpf. We then quantified global DNA methylation and the expression of the 6 *dnmt3* genes in 96 hpf larvae. The analysis was completed by measuring the expression of genes encoding for well-known stress-induced proteins, namely metallothionein (*mt2*) and heat shock proteins (*hsp70* and *hsp90*). Using genetic and epigenetic approaches, this research aimed to refine the current knowledge on the sensitivity of early embryogenesis and more precisely of the reprogramming period to environmental challenges.

## Results

### Mortality rate and hatching efficiency

**Figure 1:**
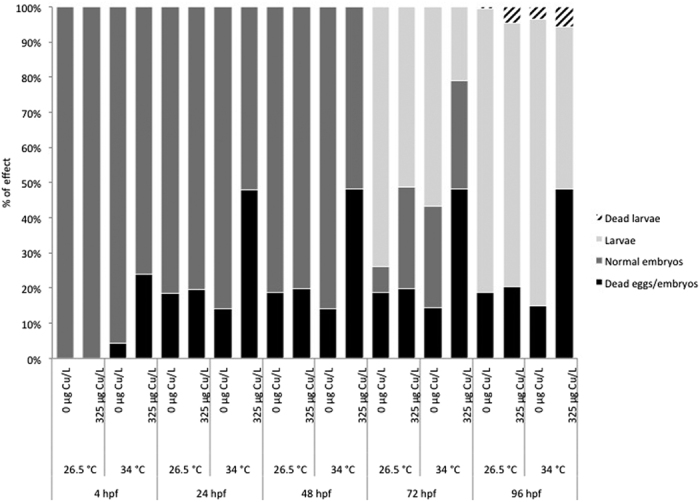
Proportion of eggs and embryos that died during the 96 hours experiment (black bars), proportion of embryos that stayed alive (dark grey bars), that hatched (i.e., larvae, light grey bars) and proportion of larvae that died (spotted dark grey bars) following exposure of zebrafish embryo from <1 to 4 hpf to 325 μg Cu/L at either 26.5 °C or 34 °C.

Figure 1 shows the proportion of eggs and embryos that died during the experiment (black bars), the proportion of embryos that stayed alive (dark grey bars), that hatched (i.e., larvae, light grey bars) and finally proportion of larvae that died (spotted dark grey bars). Mortality typically occurred within the first 24 hpf. After that period, the survival rate was stable until 96 hpf. Temperature, Cu exposure and their interaction significantly (*P* < 0.001) influenced mortality rate at 4 hpf and 24 hpf (Table 1). At 4 hpf, no mortality was recorded in groups held at control temperature, whereas we observed an increased mortality rate in groups held at 34 °C (4.4 ± 2.3% in unexposed group and 24.2 ± 7.0% in Cu-exposed group). At 24 hpf, mortality rate was increased following exposure to combined stressors with values reaching 48.0 ± 10.4%.

A peak of hatching was observed at 72 hpf in the control group (average hatching rate of 91.1 ± 3.0%), although some embryos started to hatch at 48 hpf. No interaction effect on the hatching success was observed. However, hatching rate was significantly affected at 72 hpf and 96 hpf by temperature (*P* < 0.001 at 72 hpf and *P* < 0.01 at 96 hpf) and Cu exposure (*P* < 0.001) (Table 1). Both factors significantly reduced hatching rate in an independent way. Overall, delayed embryo hatching was observed following exposure to high temperature and Cu from <1 to 4 hpf. In fact, at 72 hpf, average hatching rates of 91.1 ± 3.0% were observed in control group compared to 41.3 ± 11.2% in Cu-exposed group at 34 °C; at 96 hpf, average hatching rates of 99.1 ± 1.2% were observed in control group compared to 89.5 ± 4.6% in Cu-exposed group at 34 °C.

### DNA methylation

**Figure 2:**
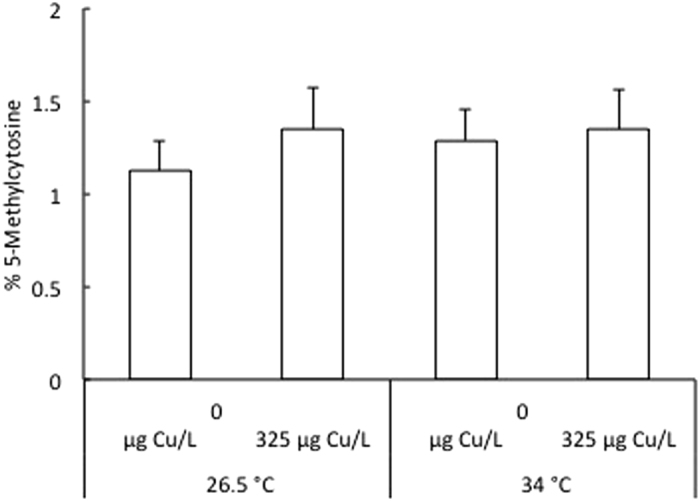
Level of methylated cytosines in 96 hpf zebrafish larvae following exposure to 325 μg Cu/L at either 26.5 °C or 34 °C from <1 to 4 hpf. Data are represented as mean ± S.E.M. (n = 6 pools, 20 embryos/pool).

The global DNA methylation levels in 96 hpf larvae following exposure to 325 μg Cu/L at either 26.5 °C or 34 °C from <1 to 4 hpf is shown in Fig. 2. The average percentage of methylated cytosines ranged between 1.12 ± 0.16% and 1.35 ± 0.21% over all treatments. There was no significant difference in global cytosine methylation levels between the four groups.

**Figure 3:**
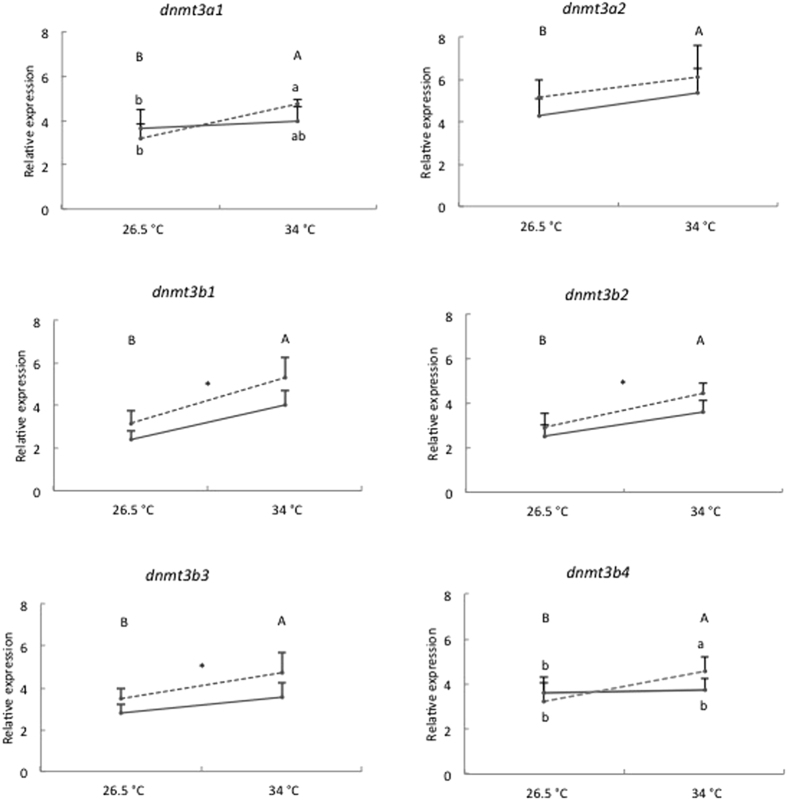
Relative expression of *dnmt3a1*, *dnmt3a2*, *dnmt3b1*, *dnmt3b2*, *dnmt3b3* and *dnmt3b4* in 96 hpf zebrafish larvae following exposure to 0 μg Cu/L (solid line) or 325 μg Cu/L (dashed line) at either 26.5 °C or 34 °C from <1 to 4 hpf. Data are represented as mean ± S.E.M (*n* = 6 pools, 30 embryos/pool). The expression is normalized to geometric mean of *ef1a* and *actb*. Different capital letters (**A,B**) indicate significant differences between temperature groups independent of copper exposure. Asterisk (*) along a line indicates a significant effect of copper exposure independent of temperature. In case of a significant interaction (dnmt3a1 and dnmt3b4) between temperature and copper exposure, different small letters (**a,b**) indicate significant differences between treatments.

The expression profiles of the 6 *dnmt3* genes in 96 hpf larvae following exposure to 325 μg Cu/L at either 26.5 °C or 34 °C from <1 to 4 hpf are depicted in Fig. 3. In our study, we followed the new nomenclature based on molecular evolution and phylogenetic analysis of *dnmt3* paralogues proposed by Campos *et al*.^28^. Temperature and Cu exposure, alone or in interaction, significantly affected the mRNA expression of *dnmt3* genes (Table 2). First, the mRNA expression of all *dnmt3* was significantly increased by exposure to high temperature, regardless of Cu exposure. The increase ranged from 22% for *dnmt3a2* and *dnmt3b4* to 68% for *dnmt3b1*. Second, the mRNA expression of *dnmt3b1*, *dnmt3b2* and *dnmt3b3* was significantly influenced by exposure to Cu, independently of heat stress. Exposure to 325 μg Cu/L significantly increased the mRNA expression of *dnmt3b1*, *dnmt3b2* and *dnmt3b3* by 33%, 20% and 29%, respectively. Finally, we observed an interaction effect of temperature and Cu exposure for the expression of *dnmt3a1* and *dnmt3b4*. Overall, their expressions were increased following exposure to both stressors. For instance, we observed an increased *dnmt3b4* expression in groups held at 34 °C (3.71 ± 0.40 in unexposed group compared to 4.56 ± 0.41 in Cu-exposed group), whereas no significant changes were recorded in groups held at 26.5 °C.

### Transcriptional expression of stress-related genes

**Figure 4:**
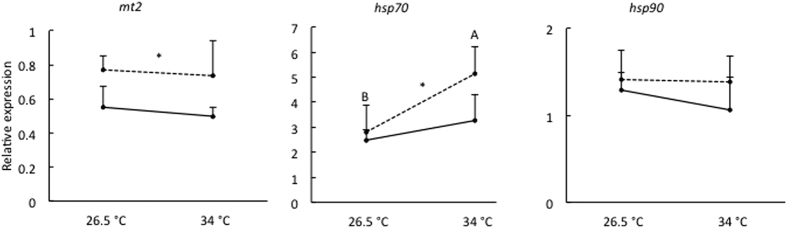
Relative expression of *mt2*, *hsp70* and *hsp90* in 96 hpf zebrafish larvae following exposure to 0 μg Cu/L (solid line) or 325 μg Cu/L (dashed line) at either 26.5 °C or 34 °C from <1 to 4 hpf. Data are represented as mean ± S.E.M. (n = 6 pools, 30 embryos/pool). The expression is normalized to geometric mean of *ef1a* and *actb*. Different capital letters (**A,B**) indicate significant differences between temperature groups independent of Cu exposure. Asterisk (*) along a line indicates a significant effect of Cu exposure independent of temperature.

The expression profiles of *mt2*, *hsp70* and *hsp90* genes in 96 hpf larvae following exposure to 325 μg Cu/L at either 26.5 °C or 34 °C from <1 to 4 hpf are depicted in Fig. 4. We did not observe any interaction between temperature and Cu exposure for the expression of studied stress genes. However, the mRNA expression of *mt2* and *hsp70* was significantly affected by Cu exposure (*P* < 0.01 and *P* < 0.05, respectively), regardless of heat stress. Exposure to 325 μg Cu/L significantly increased the mRNA expression of *mt2* and *hsp70* by 44% and 37%, respectively. Further, *hsp70* mRNA expression was significantly influenced by temperature (*P* < 0.01), independently of Cu exposure. Exposure to 34 °C significantly increased *hsp70* mRNA expression by 59%. The mRNA expression of *hsp90* was not significantly affected by temperature or Cu exposure and ranged between 1.06 ± 0.38 and 1.42 ± 0.33 over all treatments.

## Discussion

Our study was designed to investigate whether the DNA methylation reprogramming that occurs during zebrafish early development is sensitive to environmental stress factors. According to Kamstra *et al*.^7^, this is an extremely important time window to include in studies dealing with the effects of environmental challenges. Epigenetic modifications early in life are proposed to underlie the development of an adverse adult phenotype. Overall, both heat stress and copper exposure applied during reprogramming were particularly stressful and resulted in adverse developmental consequences on zebrafish, as indicated by high mortality, delayed hatching and increased expression of several stress-related genes. Further, we observed changes in the expression of *de novo* DNA methyltransferases (*dnmt3a* and *3b*) but no differences in global methylation level.

Aquatic organisms such as fish are, in most cases, exposed to multiple stressors that are either natural or anthropogenically introduced into their environment. Temperature, a potent physical stressor, and copper, a toxic chemical stressor when in excess, were addressed in the present study. Several studies have investigated the possible interaction between temperature and copper, but these were mainly conducted with adult fish. For instance, Perschbacher found an inverse relationship between temperature and copper toxicity in channel catfish, *Ictalurus punctatus*^29^. In his experiments, copper was less toxic to catfish if applied at increasing temperatures. In contrast, Furuta *et al*. observed no significant effect of temperature on copper toxicity in Japanese flounder, *Paralichthys olivaceus*, and red sea bream, *Pagrus major*^30^. More recently, Lapointe *et al*. reported synergistic effects between heat stress and copper exposure in fathead minnows *Pimephales promelas*^20^. In our study, both heat stress and copper exposure resulted in increased mortality rates and delayed embryo hatching, suggesting adverse developmental consequences on zebrafish. We highlighted increased mortality of Cu-exposed embryos with increasing temperature (significant interaction), while both stressors independently influenced the hatching success. To date, there is no study considering the interaction effects of these two stressors on zebrafish embryogenesis, although a few studies have investigated temperature effects on the toxicity of other metals, i.e., cadmium^31^ and nickel^19^. Looking first at the effects of copper, a previous study reported that exposure of zebrafish embryos to copper from fertilization to 72 hpf resulted in increased mortality between 5 and 24 hpf as well as decreased hatching success^16^. These observations are quite consistent with our results. During embryonic development, a strong influence of metals is often observed immediately after fertilization, particularly before hardening of the protective chorion. This structure is highly permeable before hardening and does not fully protect the embryo against metal penetration; thus, metals may exert direct toxic influence upon developing embryos^32^,^33^. Further, several mechanisms have been proposed to explain how copper delays the hatching process, e.g., inhibition of the hatching enzyme activity (chorionase), interference with the energy-requiring muscular movements necessary for the embryo to rupture the chorion, and alteration of oxygen uptake by the embryo^33^,^34^. However, these mechanisms are still unclear, and more than one or all of these processes may contribute to the observed delay in hatching. Secondly, zebrafish embryos normally develop within a temperature range of approximately 25–33 °C, and it was suggested that exposure to temperatures above or below these limits may produce abnormalities^35^,^36^. For instance, an increase in the developmental rates of zebrafish with increasing temperatures has been reported in several papers^37^. Schnurr *et al*. have recently assessed the thermal breadth of zebrafish embryonic development^38^. The authors observed that embryos developed successfully to hatching from 22 to 32 °C, but suffered sharp increase in mortality outside of this range. Further, the rate of embryonic development increased gradually from 22 to 34 °C, beyond which the rate dropped. Another study reported increased mortality and a higher number of developmental anomalies in embryos exposed for 2–3 h to 34–35 °C during early cleavage stages, which is similar to our results^39^.

Increasing evidence shows that environmental challenges can affect normal development of organisms by altering DNA methylation patterns. Additionally, the DNA methylation reprogramming after fertilization is a dynamic mechanism that is essential for early development and which is considered to be sensitive to environmental stressors^6^,^7^,^40^. *Dnmt3a* and *dnmt3b* are required for this process, and the inactivation of both genes causes a complete failure in the genome-wide methylation^41^. In this context, the present study investigated whether heat stress and copper exposure applied during reprogramming affect DNA methylation patterns by altering the expression of *dnmt3* genes in 96 hpf larvae. Altogether, we demonstrated that both stressors, alone or in combination, alter *dnmt3* gene expression without changes in global cytosine methylation. The absence of effects on the global scale does not mean that DNA methylation modifications do not occur. The DNA methylation assay used in this study only tested for a global shift in methylated cytosines content and does not detect changes at specific loci. This is in line with several recent studies that have reported locus-specific methylation modifications and no changes in global methylation level in zebrafish embryos^42^,^43^,^44^. For instance, Bouwmeester *et al*. observed changes in methylation levels in the promoter regions of three selected genes *vasa*, *vtg1* and *cyp19a2* but no differences in global methylation level after exposure of embryos to several environmental contaminants from 0 to 72 hpf^43^.

In addition to measuring the global DNA methylation levels, we measured the expression of all *dnmt3* genes aiming to associate their expression profiles with DNA methylation changes. Previous studies have indicated that zebrafish *dnmt3* genes exhibit distinct spatio-temporal expression patterns during embryonic development^28^,^42^,^45^,^46^,^47^. For instance, Aluru *et al*. have recently reported that *dnmt3b* genes are highly expressed in early stages of development and *dnmt3a* genes are more abundant in later stages, suggesting distinct roles for *dnmt3a* and *3b* genes during development^42^. Takayama *et al*. observed tissue-specific expression of *dnmt3a* genes during later stages of development in zebrafish, suggesting a role for these genes in tissue differentiation^47^. Other studies reported localization of *dnmt3b* genes to specific regions of the developing zebrafish, as for instance *dnmt3b1* in the aorta-gonad-mesonephros and caudal hematopoietic tissue regions^47^ and *dnmt3b2* in the brain, particularly in the tectum^3^, suggesting important roles in hematopoietic stem cell differentiation and neurogenesis, respectively. However, the specific roles of these genes in establishing *de novo* methylation patterns remain to date unknown. In the present study, we observed heat-induced upregulation of all *dnmt3* genes and copper-induced upregulation of *dnmt3b1*, *dnmt3b2* and *dnmt3b3*. Further, transcription levels of *dnmt3a1* and *dnmt3b4* were affected by copper exposure in a temperature-dependent way (significant interaction). The effects of heat stress and copper exposure on *dnmt3* gene expression levels observed in this study suggest that the establishment of DNA methylation patterns could potentially be impacted. A few recent studies have shown differences in the expression patterns of *dnmt3* genes following heat stress or pollutant exposure during zebrafish embryogenesis. Campos *et al*. observed that embryonic temperature influenced *dnmt3a* and *dnmt3b* transcript levels in a developmental stage-specific manner^28^. The authors have further reported differential responses of *dnmt3* paralogues to embryonic temperature, suggesting that they play different roles in thermal epigenetic regulation of gene expression during early development. A direct comparison with our data is tricky since the experimental conditions were different and the *dnmt3* expression during early development was reported to be very dynamic. Moreover, the oldest embryos were sampled at 80.8 hpf (protruding mouth stage) in Campos’ experiment, while we sampled zebrafish after 96 hpf. At 80.8 hpf, they didn’t observe significant differences between embryos exposed to 27 °C and 31 °C for any *dnmt3* genes. More recently, Aluru *et al*. reported that exposure to 2,3,7,8-tetrachlorodibenzo-p-dioxin (TCDD) during zebrafish early development selectively alters the expression of *dnmt* genes, and these effects were developmental stage-specific^42^. The authors also reported changes in methylation levels in promoters of aryl hydrocarbon receptor (AHR) target genes but no differences in global methylation or hydroxymethylation levels.

Metallothioneins (MTs) are low molecular weight, cysteine-rich metal-binding proteins that play essential biological roles in metal homeostasis and detoxification^48^. Heat shock proteins (HSPs) are a set of evolutionary conserved proteins that are constitutively expressed and function as molecular chaperones in protein biogenesis and homeostasis^49^. As stress response proteins, MTs and HSPs confer cellular protection against a wide variety of stress factors and can be induced at the transcription level by diverse environmental stressors, including toxic metals and temperature^50^,^51^. Moreover, previous studies suggested that MTs^52^ and HSPs^36^ play significant roles during normal embryo development in zebrafish. In our study, we observed that exposure to copper increased the transcription level of *mt2* and *hsp70* in zebrafish larvae. Concomitantly, the *hsp70* transcript level was also increased in response to thermal stress. Further, none of the applied stresses was able to affect the transcription level of *hsp90*. Similar results were previously observed in various fish species exposed either to heat or copper^20^,^53^,^54^. In zebrafish, several studies also reported that heat shock and/or copper exposure resulted in increased expression of MTs and/or HSPs in different adult tissues^55^,^56^ or in embryos^36^,^57^. Overall, our gene expression results indicate that applied conditions were stressful to zebrafish larvae, thereby suggesting the susceptibility of reprogramming period to environmental challenges.

To conclude, our study is the first to report that the DNA methylation reprogramming is sensitive to the effects of environmental stressors in zebrafish embryos. As a matter of fact, we have shown that exposure to elevated temperature and/or copper during this critical developmental period resulted in high mortality, delayed hatching and increased expression of several stress genes, i.e., *mt2* and *hsp70*. Furthermore, the expression of *dnmt3* genes suggests possible impact on the establishment of DNA methylation patterns. In the future it would be interesting to focus on DNA methylation levels of selected genes (e.g., *mt2* and *hsp70*) as well as on genome-wide methylation pattern and the relationships with gene expression. Further exploration is also needed to examine the adult phenotype of exposed embryos and to analyze molecular mechanisms that potentially link epigenetic effects and altered phenotypes.

## Materials and Methods

### Ethics statement

All zebrafish husbandry and experimental procedures were performed in accordance with the Belgian animal protection standards and were approved by the University of Namur Local research Ethics Committee (14 224 KE).

### Zebrafish embryo collection and exposure

AB line wild-type zebrafish (*Danio rerio*) were maintained in a recirculating ZebTec housing system (Techniplast) at 28 °C with a 12:12 h (light/dark) photoperiod. Conductivity was maintained at approximately 500 μS/cm, pH at 7.2 and dissolved oxygen at 95% saturation. Fish were fed *ad libitum* three times daily (twice on flake and once on *Artemia* nauplii). Males and females were placed together in breeding aquaria the night before spawning in a ratio of 3:2. The next morning, eggs were collected within 30 min of spawning, pooled from several breeding tanks, washed with ZebTec system water to remove debris and then randomly transferred into polypropylene beakers.

Zebrafish embryos were exposed to CuCl_2_ (Sigma-Aldrich 751944) at nominal concentrations of 0 and 325 μg/L from <1 hpf (before complete hardening of the protective chorion) to 4 hpf (sphere stage) at either 26.5 °C or 34 °C. The tested treatments were chosen based on previous studies^16^,^37^. Six replicates for each treatment were used and each replicate consisted of a polypropylene beaker containing 150 ml of the respective treatment solutions and 166 ± 21 embryos. After the exposure, the embryos were rinsed thoroughly with fresh ZebTec system water and incubated at 26.5 °C until 96 hpf. Half of the water in each beaker was replaced every day to remove the wastes and dead embryos. Mortality rate and hatching efficiency were recorded daily until 96 hpf. At 96 hpf, larvae were washed, collected and stored at −80 °C until DNA and RNA were isolated.

Total Cu concentration in the exposure water was measured using an Inductively Coupled Plasma-Mass Spectrometer (ICPMS, Elan DRC II). The accuracy of analytical methods was checked using certified reference standards (ICP/MS Multi-Element Standards ICP-MS-QC2-1, ACCUStandard). The mean concentration and standard deviation in the 325 μg/L treatment were 280.4 ± 13.6 μg/L.

### Global DNA methylation

Genomic DNA was isolated from zebrafish larvae (96 hpf; n = 6 pools, 20 larvae/pool) using DNeasy Blood & Tissue Kit (Qiagen) according to manufacturer’s protocol. DNA samples were treated with RNase A (Qiagen) to remove RNA contaminant. The DNA concentration and the quality of samples were assessed by spectrophotometry (NanoDrop 2000c Spectrophotometer, Thermo Scientific) and agarose gel electrophoresis. Quantification of global DNA methylation was examined by the colorimetric MethylFlash^TM^ Methylated DNA Quantification Kit (Epigentek Group Inc.) following the manufacturer’s instructions. The analysis was performed in duplicates with 100 ng of genomic DNA per sample. Absorption at 450 nm was determined. The percentage of methylated cytosines was calculated using the formula described by the manufacturer.

### Quantitative real-time PCR (qPCR)

The expression of 9 target genes was assessed by qPCR: 6 are involved in *de novo* DNA methylation (*dnmt3a1*, *dnmt3a2*, *dnmt3b1*, *dnmt3b2*, *dnmt3b3* and *dnmt3b4*) and three play important roles in cell protection against environmental stressors (*mt2, hsp70* and *hsp90*). Total RNA was isolated from zebrafish larvae (96 hpf; n = 6 pools, 30 larvae/pool) with Extract-All Reagent (Eurobio) and DNase treated (DNA-free™ Kit, Ambion) by following the manufacturers’ protocol. The RNA concentration and the quality of samples were assessed by spectrophotometry (NanoDrop 2000c Spectrophotometer, Thermo Scientific) and agarose gel electrophoresis. Complementary DNA was synthesized from 1 μg total RNA using the RevertAid H Minus First Strand cDNA Synthesis Kit (Thermo Scientific). Specific primers were selected from the literature or newly designed using Primer3Plus software and are listed in Table 3. Whenever possible, primers were designed to span at least one intron/exon border to avoid amplification of potential contaminating genomic DNA. Quantitative PCR was performed in a total volume of 20 μl (5 μl of cDNA, 2.5 μl of each primer at 5 μM, 10 μl Master Mix 2x) with SYBR Green chemistry (Applied Biosystems) on a StepOnePlus Real-Time PCR System (Applied Biosystems). The PCR conditions used were 1 cycle at 95 °C for 10 min and 40 cycles at 95 °C for 15 s and 58–61.8 °C depending on gene for 1 min (Table 3). This was followed by a melting curve analysis to verify the specificity of the PCR products. Two technical replicates were used for each sample. No reverse transcriptase and no template controls were included on each plate to ensure the absence of background contamination. Gene expression data were calculated using the relative standard curve method^58^ and normalized by geometric averaging of beta-actin (*actb*) and elongation factor alpha (*ef1a*). *actb* and *ef1a* were selected among several candidate housekeeping genes that remain stable over zebrafish development mentioned in the literature^59^,^60^, and after validation of their expression in our samples.

### Statistical analysis

Data were expressed as mean ± S.E.M. Data were log-transformed when the conditions of normality and/or homogeneity of variance were not fully filled, or arcsine-square-root-transformed if expressed in percents. Differences between groups were analyzed using two-way analysis of variance (ANOVA 2) followed by a multiple comparison Tukey’s HSD test at a 5% significant level. All tests were performed using the Statistica 5.5 software (StatSoft, Tulsa, OK, USA).

## Additional Information

**How to cite this article**: Dorts, J. *et al*. DNA methyltransferases and stress-related genes expression in zebrafish larvae after exposure to heat and copper during reprogramming of DNA methylation. *Sci. Rep.* **6**, 34254; doi: 10.1038/srep34254 (2016).

## Figures and Tables

**Table 1:** Cumulative percent mortality and hatching of zebrafish embryos/larvae following exposure to 325 μg Cu/L at either 26.5 °C or 34 °C from <1 to 4 hpf.

		26.5 °C		34 °C	
		0 μg/L	325 μg/L	0 μg/L	325 μg/L
Cumulative percent mortality	4 hpf	0.0 ± 0.0^c^	0.0 ± 0.0^c^	4.4 ± 2.3^b^	24.2 ± 7.0^a^
	24 hpf	19.0 ± 3.1^b^	19.8 ± 2.7^b^	14.0 ± ± 3.7^b^	48.0 ± 10.4^a^
	48 hpf	19.1 ± 3.0	20.0 ± 2.5	14.1 ± 3.7	48.4 ± 10.4
	72 hpf	19.1 ± 3.0	20.1 ± 2.5	14.5 ± 3.9	48.4 ± 10.4
	96 hpf	19.2 ± 2.9	20.6 ± 2.7	14.8 ± 4.3	48.4 ± 10.4
Cumulative percent hatching	48 hpf	0.7 ± 0.5	0.0 ± 0.0	0.1 ± 0.2	0.1 ± 0.2
	72 hpf	91.1 ± 3.0	65.2 ± 10.0	65.6 ± 14.1	41.3 ± 11.2
	96 hpf	99.1 ± 1.2	93.8 ± 2.4	95.9 ± 2.2	89.5 ± 4.6

Data are represented as mean ± S.E.M. (*n* = 6).

Different letters (a–c) mean significant (*P* < 0.001) interaction effect between treatments within a given time period.

**Table 2:** Two-way ANOVA performed for relative expression of *dnmt3* genes in 96 hpf zebrafish larvae following exposure to 325 μg Cu/L at either 26.5 °C or 34 °C from <1 to 4 hpf, testing the effect of temperature treatment and copper exposure.

	Temperature	Copper	Temperature × Copper
*dnmt3a1*	*P* < 0.01	*P* > 0.05	*P* < 0.05
*dnmt3a2*	*P* < 0.05	*P* > 0.05	*P* > 0.05
*dnmt3b1*	*P* < 0.001	*P* < 0.001	*P* > 0.05
*dnmt3b2*	*P* < 0.001	*P* < 0.01	*P* > 0.05
*dnmt3b3*	*P* < 0.01	*P* < 0.01	*P* > 0.05
*dnmt3b4*	*P* < 0.05	*P* > 0.05	*P* < 0.05

Given are *P* values of each gene.

**Table 3:** List of specific primers used for quantitative real-time PCR.

Gene	Accession		Primer (5′–3′)	Size (bp)	*E* (%)	Ta	Reference
*dnmt3a1*	AB196917	F	GCTAAGTTTGGTAAAGTGCGG	103	96	58 °C	28
		R	GGATGTCCTCCTTATCATTCA				
*dnmt3a2*	AB196919	F	TAGGAAAGGCTTGTTTGAGGG	157	105	59 °C	28
		R	GCGTGAGATGTCTTTCTTGTC				
*dnmt3b1*	AB196915	F	GTGCCTCTGGGATGGATAAA	174	98	60 °C	Newly designed
		R	GCTCTGTGCACCACAGGATA				
*dnmt3b2*	AB196918	F	CGCTACATTGCCTCTGAGA	113	95	58 °C	28
		R	GCCAGATGTTTCCTAGTGATG				
*dnmt3b3*	AB196914	F	GCTCAGGTGCTGCTTTTTGTC	152	95	61 °C	28
		R	TTTTTGAATCTGTGCTTTGCTG				
*dnmt3b4*	AB196916	F	TCATTGACTTGGGGGAAGAG	172	97	60 °C	Newly designed
		R	TAATCATCATGGCTGCTGCT				
*mt2*	BC152694	F	AAGCTCTTTGTGGATACTCTCTGG	112	94	60.8 °C	Newly designed
		R	GCAGGTAGTACACTGGCAATTAGTG				
*hsp70*	AB062116	F	CATCGACGCCAACGGG	191	95	60 °C	61
		R	CCAGGGAGTTTTTAGCAGAAATCTT				
*hsp90*	BC075757	F	GGTACCAAAGTCATTCTCCACCTT	154	97	61.8 °C	Newly designed
		R	CCTCAAGATCCACCTCTTTTTCTC				
*actb*	BC165823	F	CCAACACTGTATTGTCTGGTGGTA	203	101	61 °C	Newly designed
		R	GTACTCCTGCTTGCTAATCCACAT				
*ef1a*	AY422992	F	GGTACTACTCTTCTTGATGCCCTTG	200	98	60.8 °C	Newly designed
		R	GACTTGACCTCAGTGGTTACATTG				

For each gene, its GenBank accession number, amplicon size (bp), amplification efficiency (E) and annealing temperature (Ta) are indicated.
